# Cochrane Interactive Learning

**DOI:** 10.5195/jmla.2018.516

**Published:** 2018-10-01

**Authors:** Kate Ghezzi-Kopel

**Affiliations:** Albert R. Mann Library, Cornell University, Ithaca, NY

## INTRODUCTION

Cochrane Interactive Learning (CIL) is a modular, self-directed, educational tool for researchers performing systematic reviews. With the volume of published and unpublished biomedical evidence growing exponentially each day [1], systematic reviews are critical tools for informing clinical practice and decision-making. Both new and experienced researchers must adhere to a rigorous set of standards to produce unbiased and transparent systematic reviews, and robust training is necessary to learn the complex requirements of the methodology. CIL provides an accessible, well-designed, step-by-step guide for navigating the systematic review process.

Users of CIL immerse themselves in systematic reviews by completing nine multimedia modules. Each module consists of animations, videos, interactive exercises, and readings. Systematic review authors following the Cochrane methodology are the primary audience of CIL, and even authors undertaking non-Cochrane systematic reviews will benefit from the array of materials that are provided. Cochrane authors have free access to all CIL modules, while non-Cochrane authors can purchase the full course at a standard rate [2]. Cochrane members receive a 45% discount on the standard rate, and module 1 is free for all registered users. CIL covers a wide range of topics from writing systematic review protocols to preparing and disseminating the published systematic review.

CIL is also relevant to librarians and information specialists who collaborate on systematic reviews. Townsend et al. have developed a competency framework for librarians who are involved in systematic reviews, and many of these competencies are covered in detail in the CIL modules [3]. Librarian tasks that are identified for systematic reviews include protocol development, research question refinement, literature searching, and data management, among others. Module 3: “Searching for Studies” offers particularly relevant topics for librarians, including a clearly presented video on how to use Boolean operators.

## OVERVIEW

CIL was developed by the Cochrane Learning and Support Department in collaboration with multiple e-learning partners [4]. All of the modules are in accordance with the *Cochrane Handbook for Systematic Reviews of Interventions* and the *Methodological Expectations of Cochrane Intervention Reviews.* Completion of all nine modules will take more than ten hours on average, and the content is monitored by Cochrane staff and updated regularly. It was updated in January 2018, and another update was scheduled for July 2018 [4].

The CIL modules are divided into multiple sections, and each module opens with a guide to content navigation. A filtering tool sorts content by status or topic, and a status bar at the top of each section tracks progress. A “Cochrane Tips” icon appears next to content that is specific to Cochrane Reviews and links to a pop-out box with more information. A “Resources” button links to relevant content in the *Cochrane Handbook for Systematic Reviews of Interventions* [5].

At the end of each module, there is a self-assessment that includes multiple choice and fill-in-the-blank questions based on the learning outcomes for that module. Users are given two attempts to answer each question correctly before they are directed back to the appropriate page to revisit their learning in that area [4]. A personalized certificate is available for download after successful completion of each assessment.

## FEATURES OF THE MODULES

The first of the nine multimedia modules is a broad introduction to systematic reviews. It begins with videos and drop-down panels that illustrate key features of systematic review methodology. The module covers various types of review questions, and explains the population, intervention, comparison, outcome, study design (PICOS) question framework. One exercise asks participants to estimate the time that it takes to do a systematic review, the typical number of authors on a team, and the average amount of studies that are included in a systematic review.

Module 1 provides a high-quality interactive graphic depicting the steps in the systematic review process. The content in this first module orients researchers and librarians to the structure of systematic reviews.

Module 2 explains the rationale for creating a systematic review protocol and writing eligibility criteria based on a PICOS question, and the importance of adherence to systematic review protocols to prevent the introduction of bias.

Search strategy development is a fundamental piece of the systematic review process. Module 3 discusses the necessary rigor of systematic review searches, the importance of searching multiple databases, and how to structure searches effectively. It also covers study design filters, Boolean operators, citation management, search documentation, and strategies for updating the search.

Module 3 highlights the importance of working with an information specialist, but it does not mention that librarians frequently take on additional roles in the systematic review process, often serving as coauthors. In addition to writing the search strategy, librarians can help develop the protocol, lead citation management, guide article screening, and write the methods section for the resulting article. A separate section on working with a librarian would be a helpful addition to module 3.

Modules 4, 5, and 6 present methods for approaching study selection, risk-of-bias assessment, and data analysis. Module 4 discusses the Preferred Reporting Items for Systematic Reviews and Meta-Analyses (PRISMA) flow diagram, a chart that depicts the flow of information through the different phases of a systematic review [6], and its role in documentation and transparency. Module 5 addresses the need for a well-performed risk-of-bias assessment to support the strength of the systematic review recommendations. Module 6 provides a complete description of how to analyze different types of data and perform a meta-analysis. This section is comprehensive and could serve as a stand-alone resource for researchers performing statistical analysis.

Module 7 covers interpretation of statistical analysis, reporting bias, overall certainty of evidence, and tips for representing these items in a systematic review protocol. Module 8 explains how to prepare the summary of findings table and the abstract of a Cochrane review. Finally, Module 9 discusses how to present systematic review findings to stakeholders for implementation into practice. This section focuses on decision algorithms and frameworks for economic principles and evaluations in Cochrane reviews.

## QUALITY OF CONTENT

CIL is an invaluable tool for Cochrane authors, assuming that they have some background in clinical practice, study design, and systematic reviews. The modules include many real-world examples and multi-format exercises that allow users to apply their new knowledge. The design of the platform is clean and easy to navigate.

A potential concern is the high level of difficulty of several of the self-assessments. Detail and specificity are crucial to the systematic review process, but exercises such as parsing out the PICOS elements of a complex clinical question, for example, may be difficult for first-time reviewers. Systematic review authors who are unfamiliar with systematic review methodology may find it challenging to make the distinction between Cochrane-specific requirements and standard requirements that are presented in the learning modules. Using the “Cochrane Tip” icon is often effective in clarifying this distinction. Librarians using CIL to answer systematic review questions will also need to consider the differences between Cochrane and non-Cochrane reviews.

CIL also lacks discussion of other types of evidence synthesis. As systematic review methodology is adopted across environments and disciplines, users would benefit from contextual presentation of other methodologies, such as rapid reviews, scoping reviews, and systematic maps.

## COMPARISON TO OTHER PRODUCTS

There are several massive open online courses (MOOCS) on systematic reviews, such as Coursera’s Introduction to Systematic Review and Meta-Analysis [7], and proprietary workshops developed at individual institutions to teach systematic review methodology. Aside from these resources, there are no other online, modular, interactive teaching tools for performing systematic reviews that exist at the level of depth and specificity of CIL to this reviewer’s knowledge.

## VALUE FOR COST

For institutions where production of Cochrane reviews is common and where the library has an investment in systematic review teaching and collaboration, the value for the cost of CIL is considerable. In addition, CIL’s discount pricing options support efforts to provide systematic review training for researchers and information specialists in resource-limited settings.

## CONCLUSIONS

Despite reservations about the high level of difficulty for beginners, CIL offers robust features, granular detail, and comprehensive assessment options for researchers and librarians who are involved with systematic reviews. It is a much-needed resource for Cochrane and non-Cochrane systematic review authors alike.
